# Useful Reduction Mammoplasty Technique in Oncoplastic Breast Surgery and Reconstruction

**DOI:** 10.1155/2022/2952322

**Published:** 2022-10-21

**Authors:** Jong Ho Lee, Jeong Yeop Ryu, Kang Young Choi, Jung Dug Yang, Ho Yun Chung, Byung Chae Cho, Byeongju Kang, Jeeyeon Lee, Ho Yong Park, Joon Seok Lee

**Affiliations:** ^1^Department of Plastic and Reconstructive Surgery, School of Medicine, Kyungpook National University, Daegu, Republic of Korea; ^2^Department of Surgery, School of Medicine, Kyungpook National University, Kyungpook National University Chilgok Hospital, Daegu, Republic of Korea

## Abstract

**Background:**

A combination of the reduction mammoplasty technique and breast reconstruction allows surgeons to lift ptotic breasts through local flaps and skin reduction during surgery for breast cancer. This study presents a reliable course for the combination of partial and skin or nipple-sparing mastectomy with reduction-reconstruction surgery.

**Methods:**

Fifty-seven patients underwent a partial mastectomy before reduction mammoplasty of both breasts during the same time period between 2014 and 2021 at our institution and thirteen patients underwent skin or nipple-sparing mastectomy, breast reconstruction with an extended latissimus dorsi flap or silicone implant, and aesthetic reduction mammoplasty of the contralateral breast during the same time period. Additional photos were obtained preoperatively, immediately after the operation, and at one, three, six, and twelve months postoperatively. Patient satisfaction was evaluated preoperatively and postoperatively and postoperative complications were noted.

**Results:**

Among the patients who underwent a partial mastectomy, the mean age was 45.18 ± 11.05 years, the mean body mass index (BMI) was 26.74 ± 3.53 kg/m^2^, and the mean preoperative right and left breast volumes were 663.85 (±28.12) cc and 664.34 (±37.13) cc, respectively, and the mean excised mass weight was 177.74 (±213.93) g. Among the patients who underwent a skin-sparing mastectomy, the mean age was 51.62 ± 8.96 years, the mean BMI was 26.91 ± 4.34 kg/m^2^, and the mean preoperative right and left breast volumes were 624.17 (±98.52) cc and 562.31 (±80.81) cc, respectively, and the mean excised mass weight was 618.05 (±338.17) g. Four patients (5.3%) in the partial mastectomy group had fat necrosis. The mean patient satisfaction score was higher postoperatively in both groups.

**Conclusion:**

Patients with breast cancer and large and/or ptotic breasts can successfully undergo reduction mammoplasty for both breasts immediately following partial mastectomy and nipple or skin-sparing mastectomy.

## 1. Introduction

Breasts are anatomically and symbolically important for women. In breastfeeding women, the breast parenchyma expands and naturally shrinks after the lactation period; however, the stretched skin may not shrink, resulting in patient discomfort. Breast ptosis becomes more severe when combined with the natural aging process, leading to cosmetic or daily life discomfort [1]. When breast cancer requires surgical treatment, whether to perform a partial or total mastectomy is determined based on the early diagnosis and the extent of cancer. The surgical method and the amount of excision affect the possible reconstruction methods [2].

Oncoplastic breast reductions allow wide resections with free margins and can be used for large cancers as an alternative to mastectomy [3]. Typically, more breast reconstruction methods are available for patients with large or ptotic breasts, including oncoplastic breast surgery, using reduction mammoplasty or mastopexy [4, 5]. While Asian females tend to have smaller breast sizes and less severe ptosis than Westerners, recent changes in body composition due to Westernized eating habits and lifestyles have resulted in an increased number of patients with congenitally large breasts [6]. For these patients, the breasts can be lifted during cancer surgery via a local flap and skin reduction, and the breast parenchyma tissue is preserved after breast resection using a combined reduction mammoplasty and breast reconstruction technique [7]. In patients undergoing a nipple or skin-sparing mastectomy (NSSM), it can be performed in combination with breast cancer surgery to obtain a more favorable breast contour by reducing the total volume of the breast as much as possible, increasing the projection of the ptotic breast, and restoring the volume of the upper pole. Despite the considerable scar length, patient satisfaction after surgery is very high [8]. Furthermore, a more favorable breast shape can be obtained during cancer treatment than during simple breast reconstruction for restoring appearance before surgery and may provide psychological assistance to endure cancer treatment [9]. While reduction mammoplasty is typically performed through an inverted T incision, other techniques are useful based on the location of the breast cancer or the pedicle. However, there is a lack of established management for cosmetically matching the size and shape of the contralateral breast after performing breast reduction for reconstruction after mastectomy in large or ptotic breasts, especially when cancer is located beside the reduction design; this study presents a method applicable to patients undergoing partial and nipple or skin-sparing mastectomy with reduction mammoplasty surgery.

## 2. Methods

**Figure 1:**
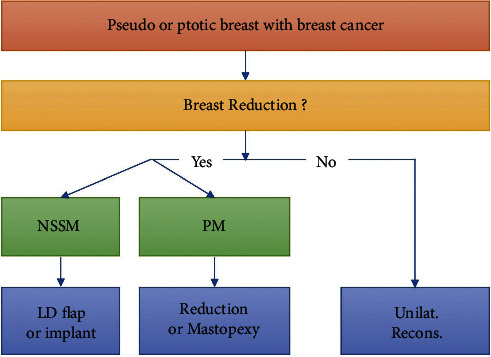
Algorithm for reconstructive breast surgery in true ptotic or pseudoptotic breasts. Patients with breast cancer with ptotic breasts who desired breast reconstruction underwent a nipple or skin-sparing mastectomy (NSSM) using a latissimus dorsi (LD) flap or implant or a partial mastectomy (PM) with reduction or mastopexy. Patients that did not desire breast reconstruction underwent unilateral reconstruction (unilat. recons.) and were not included in this study.

**Figure 2:**
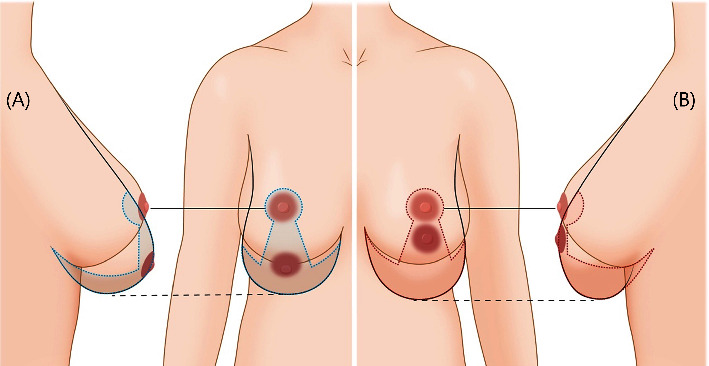
Concept illustration in ptotic and pseudoptotic breasts. The wise pattern of inverted T reduction mammoplasty is applicable in patients with (a) ptotic or (b) pseudoptotic breasts. However, customized designs are necessary for different skin excision areas, vertical line lengths, NAC locations, and lower pole shapes. NAC, nipple-areolar complex.

This retrospective, single-center study included 70 Korean patients who underwent oncoplastic breast reconstruction via the reduction mammoplasty technique between April 2014 and November 2021. The study protocol was approved on February 22, 2022, by the Institutional Review Board (KNUCH2022-02-002) at the Kyungpook National University Chilgok Hospital. Patients with true or pseudoptotic breasts who wished to undergo mastopexy or reduction during breast cancer surgery were included in this study (Figures 1 and 2).

**Figure 3:**
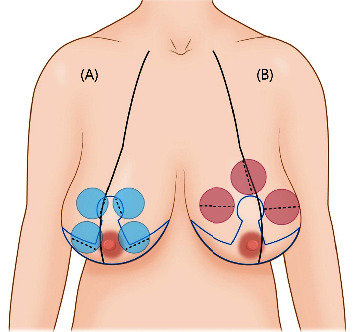
Incision location based on cancer location and reduction mammoplasty design. (a) When the breast cancer was located within the reduction mammoplasty design, the incision was made on the inner area of the design (dotted line) to prevent additional scarring. (b) When the breast cancer was located outside of the reduction mammoplasty design, the incision was made at the midline of the cancer locations.

Fifty-seven patients underwent a partial mastectomy (PM) based on the cancer location and bilateral reduction mammoplasty and thirteen patients underwent nipple or skin-sparing mastectomy (NSSM), breast reconstruction with an extended latissimus dorsi (LD) flap or silicone implant, and aesthetic reduction mammoplasty on the contralateral breast to achieve breast symmetry. PM was performed by a breast surgeon after consulting with a plastic surgeon. The location and shape of the incision were based on the reduction mammoplasty design. If the cancer was located within the reduction mammoplasty design, the incisions were made on the excision area, that is, on the inner side of the design to prevent future scarring. If the cancer was not located within the reduction mammoplasty design, the incisions were made on the midline of the cancer locations to optimize tissue removal (Figure 3). Then, sentinel lymph node biopsy or axillary lymph node dissection was performed via a 3 F02D 4 cm incision in the axillary area. Photos of the patient were obtained preoperatively, immediately after the operation, and at one, three, six, and 12 months postoperatively.

### 2.1. Design of Reduction Mammoplasty Technique

**Figure 4:**
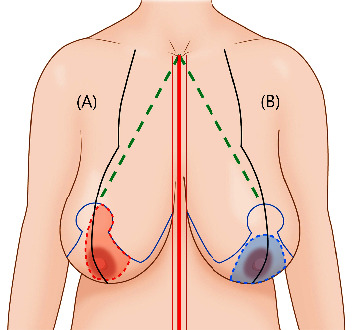
Preoperative design and pedicle selection in reduction mammoplasty using the inverted T technique. (a) The superomedial pedicle design is typically used in patients with an intact blood supply on the nipple-areolar complex. (b) In patients with breast cancer adjacent to the superomedial pedicle, the inferior pedicle design is used. The distance between the sternal notch and the upper margin of the neo-nipple-areolar complex is 18–21 cm (green dotted line). The thick red line represents the midline, and the thin red lines represent skin tissue margins.

Reduction mammoplasty is designed for patients who desire breast reconstruction during breast cancer surgery. In the upright position, the standard is set with the lower margin of the new areola located at the breast mound in the projective position at the inframammary fold (IMF). The design is planned using an omega-shaped marker. The distance between the sternal notch and the final areola location is marked at 18–21 cm, based on the patient's body mass index (BMI). A 1 cm margin is maintained on both sides of the midline to avoid an unnatural intersection of the bilateral IMF incisions. The lateral margin is designed to result in a rounded shape based on the lateral breast volume. The two oblique lines of the wise pattern are approximately 6–8 cm, and the contour and the amount of resection are assessed by temporary stapling. The angle formed by the two oblique lines is 60–80° in case breast width reduction is required. When only improvement in projection and repositioning of the nipple-areolar complex (NAC) is required, then the angle formed by the oblique lines is 30–60°. The pedicle is typically designed as a superomedial pedicle to facilitate the correction of the IMF and the repositioning of the NAC. When the cancer location is close to the superomedial pedicle, then the inferior pedicle is used (Figure 4).

### 2.2. Operative Technique

#### 2.2.1. Partial Mastectomy (PM) and Oncoplastic Breast Surgery with Reduction Mammoplasty

During the planning of partial mastectomy surgery, a reduction mammoplasty is designed by a plastic surgeon who is also consulted regarding the operative incision. Mass excision is performed while maintaining the breast skin flap without damaging the selected pedicle toward the inner side of the design when the cancer is located within the design. The amount of resection of the skin flap is adjusted based on the excised volume by the breast surgeon; however, if the cancer is not within the reduction mammoplasty design, an incision is made in the middle of the mass in the radial incision direction from the nipple. After reshaping and elevating the glandular flaps bilaterally by the plastic surgeon, closure is performed, and modifications are conducted based on the excised mass volume to achieve the preoperative design. When bleeding is controlled, temporary stapling is performed in the sitting position to adjust the final shape of the breast before contralateral reduction mammoplasty is performed. The intersection of the bilateral oblique lines, which forms the vertical scar, is balanced with the neo-IMF and the neo-nipple. The diameter of the new areola is typically 38–40 mm, based on the patient's BMI and the size of the original nipple.

#### 2.2.2. Nipple or Skin Sparing Mastectomy (NSSM) and Breast Reconstruction with Extended LD Flap or Silicone Implant

During the planning of nipple or skin-sparing mastectomy surgery, a reduction mammoplasty is designed by a plastic surgeon who is also consulted regarding the operative incision. The blood supply is preserved superior to the NAC via the vasculature of the skin flap identified on a preoperative 3D-reconstructed breast magnetic resonance image [10–12]. The inner area of the design is marked with a 5–8 cm, vertical, straight line to preserve the blood supply of the NAC as much as possible. Mastectomy and sentinel lymph node sampling are performed, and the patient is moved from the decubitus position to prepare for the extended LD flap. Although the breast volume is reduced during mastectomy, the skin paddle should be designed using the smallest LD flap as possible. The extended LD flap is elevated using an electrical device to achieve full detachment of the humoral attachment to secure the flap length and prevent difficulty in transferring it to the breast defect. To prevent future jerking movements, the thoracodorsal nerve is cut, and the main pedicle, along with the thoracodorsal artery and vein, is transported to the breast via axillary tunneling without twisting or folding. Then, a negative drain is inserted, fibrin glue spray is used, and a layer-by-layer suture is performed. The patient is then moved to the sitting position, and a bilateral reduction mammoplasty is conducted. Stapling is performed according to the preoperative design, and the shape and size of the breast that has undergone NSM are measured. Symmetry in the shape and size of the breast is achieved by excising tissue based on the amount excised and the extended LD flap used in the first breast. In the case of implant insertion instead of LD flap, the size and shape are selected considering the symmetry of both breasts. To preserve blood flow to the NAC, the mastectomy skin flap must not be damaged via tension or incision. Full-thickness skin incisions should be avoided during reductions, and the subdermal plexus should be preserved as much as possible via deepithelialization [13].

### 2.3. Data Collection and Analysis

Patient age, BMI, cancer stage, breast surgery, and reconstruction method were recorded. Data regarding breast symmetry were collected by analyzing the profile (projection and width) of the reconstructed nipple in photos obtained preoperatively and at three, six, and 12 months postoperatively. The bilateral nipples were compared. A questionnaire regarding preoperative and postoperative patient satisfaction using a visual analog scale (VAS) score of 1–5 (1 representing low satisfaction and 5 representing high satisfaction) extracted from the Breast-Q was created for this study and was conducted one year after surgery.

### 2.4. Statistical Analysis

All statistical analyses were conducted using SPSS Statistics for Windows, version 16.0 (SPSS Inc., Chicago, Ill., USA). Data were compared using the paired *t*-test. Statistical significance was set at *P* < 0.05.

## 3. Results

Among the 57 patients who underwent a partial mastectomy, the mean age was 45.18 ± 11.05 years, the mean BMI was 26.74 ± 3.53 kg/m^2^, and the mean preoperative right and left breast volumes were 663.85 (±28.12) cc and 664.34 (±37.13) cc, respectively, and the mean excised mass weight was 177.74 (± 213.93) g. Among the 13 patients who underwent NSM, the mean age was 51.62 ± 8.96 years, the mean BMI was 26.91 ± 4.34 kg/m^2^, and the mean preoperative right and left breast volumes were 624.17 (±98.52) cc and 562.31 (±80.81) cc, respectively, and the mean excised mass weight was 618.05 (±338.17) g. Ductal carcinoma in situ (DCIS) was identified in 14 (24.6%) patients in the partial mastectomy group and two patients (23.1%) in the NSM group. Forty-two (73.7%) patients in the partial mastectomy group and 10 (76.9%) patients in the NSM group were found to have invasive ductal carcinoma (IDC). Mucinous carcinoma was present in 1 patient (1.8%) in the partial mastectomy group. Additionally, in the partial mastectomy group, 18 (31.6%) patients had stage 0 cancer, 22 (38.6%) patients had stage I cancer, and 3 (5.3%) patients had stage II cancer. In the NSM group, 3 (23.1%) patients had stage 0 cancer, 5 (38.5%) patients had stage I cancer, and 2 (15.4%) patients had stage II cancer. No patient was diagnosed with stage III or IV cancer in either group (Table 1). Inferior pedicles were used in 5 (8.8%) patients in the partial mastectomy group due to the location of cancer. In the NSM group, an LD flap with an implant was used in 3 (23.1%) patients, an LD flap in 8 (61.6%) patients, and an implant only in 2 (15.9%) patients.

Six (10.5%) patients in the partial mastectomy group and 3 (23.1%) patients in the NSM group experienced seroma. Minor T junction necrosis was identified in 5 (8.8%) patients in the partial mastectomy group and 4 (30.1%) patients in the NSM group. Major T junction necrosis occurred in 3 (5.3%) patients in the partial mastectomy group and 2 (15.4%) patients in the NSM group. Partial NAC necrosis occurred in 3 (5.3%) patients in the partial mastectomy group and 3 (23.1%) patients in the NSM group, and total NAC necrosis occurred in zero patients in the partial mastectomy group and 3 (23.1%) patients in the NSM group. Fat necrosis occurred in 4 (7.0%) patients in the partial mastectomy group and zero patients in the NSM group. Hematomas occurred in 3 patients (5.3%) in the partial mastectomy group and 3 (23.3%) patients in the NSM group. No patients had an infection (Table 2). Among these items, seroma, NAC necrosis, and infection had statistical significance.

### 3.1. Outcome Evaluation

Patient satisfaction regarding breast symmetry improved from 3.9 ± 0.4 points preoperatively to 4.0 ± 0.2 postoperatively. Patient satisfaction regarding breast shape improved from 3.2 ± 0.3 points preoperatively to 4.1 ± 0.2 points postoperatively, and patient satisfaction regarding NAC shape improved from 3.5 ± 0.1 points preoperatively to 4.0 ± 0.3 points postoperatively. In terms of psychosocial well-being, patient satisfaction regarding natural breast contour improved from 3.5 ± 0.2 points preoperatively to 4.3 ± 0.1 points postoperatively, patient satisfaction regarding comfort improved from 3.4 ± 0.4 points preoperatively to 4.2 ± 0.4 points postoperatively, and patient satisfaction regarding clothing improved from 3.3 ± 0.3 points preoperatively to 4.3 ± 0.3 points postoperatively. In terms of sexual well-being, patient satisfaction regarding confidence improved from 3.6 ± 0.1 points preoperatively to 3.9 + 0.2 points postoperatively and patient satisfaction regarding mirror image improved from 3.5 ± 0.3 points preoperatively to 4.0 ± 0.1 points postoperatively. All satisfactory outcome data were statistically significant, except for “bra comfort” and “attractiveness in the mirror” items (Table 3).

## 4. Discussion

Mastectomy is an essential part of the treatment of breast cancer and is classified into partial and total depending on the resection scope. Recently, as oncological safety has been confirmed, the number of cases in which breast reconstruction is simultaneously performed with mastectomy is increasing [14]. While filling in the total or partial defect caused by the excision of breast cancer is the basic concept of reconstruction, there has been a recent growth in preference related to body image [15]. With the development of early breast cancer diagnosis technology and the rise in the socioeconomic status of patients, options that can be helpful in the quality of life after treatment rather than operations only for cancer treatment are being selected [16]. Based on this trend, an increase in satisfaction with the procedure in which the shape of the breast is resolved in the direction of enhancing its beauty, rather than simply restoring the preoperative body image, has been observed [17]. Therefore, for patients diagnosed with breast cancer who had considered reduction mammoplasty before being diagnosed with breast cancer or patients concerned about ptotic or flattened breasts with decreased elasticity, a reduction mammoplasty or mastopexy operation may be warranted during cancer treatment. The concurrent performance of breast reconstruction and total or partial mastectomy to achieve a desirable bilateral breast shape through volume reduction and breast lifting is a viable option even for patients with unilateral cancer [18]. However, whether to perform a partial mastectomy or a total mastectomy is determined depending on the location and extent of the breast cancer. Although the reduction mammoplasty algorithm for large or ptotic breasts has been established, its combination with breast cancer surgery has not. In this study, an algorithm for the treatment of patients who are undergoing mastectomy due to breast cancer and desire concurrent breast reduction or mastopexy is presented.

**Figure 5:**
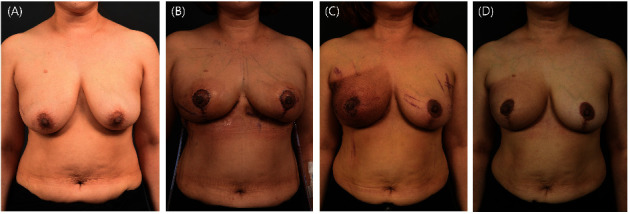
Partial mastectomy and reduction mammoplasty in a patient with right breast cancer. A patient with a breast cancer tumor in the right lower quadrant of the right breast, located far from the Wise pattern reduction mammoplasty design, underwent glandular reshaping and reduction mammoplasty followed by contralateral reduction mammoplasty for asymmetry. (a) Preoperative image. (b) Five days postoperative image. (c) Two months postoperative image. The patient had been undergoing adjuvant radiotherapy. The skin tanning was treated with moisturizer. (d) Fourteen months postoperative image. The tanning had healed and the patient reported satisfaction.

**Figure 6:**
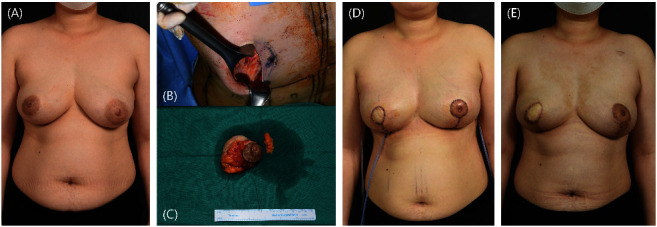
Partial mastectomy and reconstruction mammoplasty of patients with centralized right breast tumor. A patient with a tumor involving the nipple-areolar complex (NAC) of the right breast underwent tumor reduction mammoplasty with a superomedial pedicle. The deepithelized inferior tissue was transpositioned to the neo-NAC area for delayed NAC reconstruction. (a) Preoperative image. (b, c) The defect and the mass are shown intraoperatively. (d) Five days postoperative image. (e) One year postoperative image.

**Figure 7:**
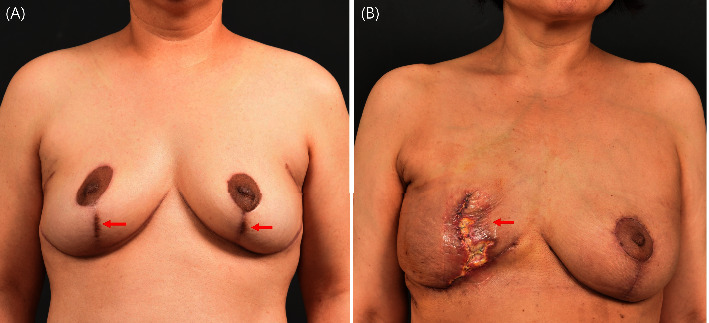
Postoperative complications. (a) Remnant areolar tissue is observed on the postoperative vertical scar of both inferior poles. (b) Skin-sparing mastectomy and extended latissimus dorsi flap reconstruction resulted in skin flap necrosis with a poor aesthetic outcome due to insufficient blood supply from the subdermal plexus injury.

Various techniques have been reported for the reduction mammoplasty [19], including the inverted T reduction mammoplasty technique using the superomedial or inferior pedicle. This technique allows for the adaptation of the width, projection, and height of the breast [20–22]. This combination procedure can be performed in patients with breast cancer and moderate to large or ptotic breasts who desire reduction or mastopexy. When a partial mastectomy is conducted, an inverted T reduction mammoplasty can be designed with the assistance of a breast surgeon. After confirming the location and extent of cancer preoperatively using sono-guided marking, the incisions are planned according to the reduction mammoplasty design and cancer location. When the cancer is located within the area to be excised during reduction mammoplasty, the incision is included inside the excised area to reduce the number of scars. However, a radial incision centered on the nipple is used when the cancer is not within the excision area. Once an intraoperative frozen biopsy is confirmed to be negative, the reduction mammoplasty design can be corrected and glandular rearrangement of the area can occur to achieve more reliable and favorable outcomes. Matching the defect to the breast contour is challenging even when the cancer is thoroughly excised; mismatching results in depressions. Therefore, the patient must be thoroughly counseled preoperatively, and the symmetry of the breasts should be evaluated thoroughly during surgery. If a long vertical scar forms, pseudoptosis may occur, requiring additional revision operations [23]. As most procedures can be conducted using the superomedial pedicle, complications such as ischemia or necrosis on the NAC side are rare [24]. In this study, when patients had cancer in the superomedial pedicle, the inferior pedicle was used during partial mastectomy (Figure 2). After surgery, radiation and chemotherapy were performed without any major problems, and patient satisfaction was maintained fairly well after more than one year (Figure 5). When patients in this study had cancer in the central region, the breast was excised around the NAC, and matching the symmetry of the entire breast via significant advantages in later NAC reconstruction via transpositioning the skin of the inferior part to be deepithelized was achieved (Figure 6). However, some patients with moderate ptosis and large areolar areas had areolar tissue remaining in the vertical scar (Figure 7(a)). This should be considered during preoperative planning and must be avoided during surgery.

**Figure 8:**
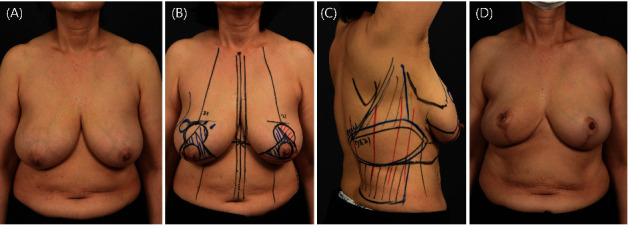
Reduction mammoplasty and nipple or skin-sparing mastectomy (NSSM) in patients with right breast cancer. A patient with a breast cancer tumor in the right upper quadrant of the right breast adjacent to the reduction mammoplasty design is shown. An extended latissimus dorsi (LD) flap with a 7 × 21 cm skin paddle was designed. The excised tissue weight in contralateral reduction mammoplasty was 341 g. (a) Preoperative image. (b, c) Preoperative reduction mammoplasty and extended LD flap designs. (d) One year postoperative image.

**Figure 9:**
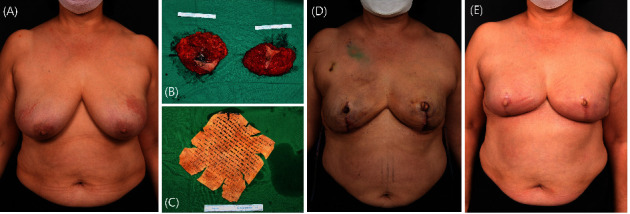
Implant-based breast reconstruction after skin-sparing mastectomy. A patient with bilateral breast cancer underwent bilateral skin-sparing mastectomy followed by bilateral reduction mammoplasty. Megaderm (L&C BIO Inc., Seoul, Korea) (20 × 20 cm, 1.5–2 mm thick) was covered on a prepectoral plane-breast silicone implant using the anterior coverage technique. (a) Preoperative image. (b, c) The excised masses and acellular dermal matrix (ADM) are shown intraoperatively. (d) Three days postoperative image. (e) Eight months postoperative image.

During total mastectomy surgeries, the blood supply must be considered carefully. The possibility of partial or total necrosis of the breast skin flap and NAC must be evaluated preoperatively. [25] During reduction mammoplasty with an extended LD flap or silicone implant, the skin flap incision of a large breast that undergoes NSM must not be in full depth to avoid damage to the blood vessels connected to the NAC, which would increase the probability of necrosis. [26, 27] Therefore, only delicate deepithelization should be performed to minimize the damage [28, 29]. Seroma and NAC necrosis occurred more often after total mastectomy than after partial mastectomy in this study with statistical significance (Table 2). As for the categories, complications in the minor and partial mild groups were cases where wound healing was completed only with conservative management, and in the major and total groups, a revisional operation was required. Several complications often occurred concomitantly in individual patients, especially in patients who underwent skin-sparing mastectomy (SSM) in this study (Figure 7(b)). When reduction mammoplasty using an LD flap was conducted during reconstruction after a mastectomy, fairly satisfactory results were achieved using autologous tissue (Figure 8). A breast surgeon was consulted preoperatively to plan the surgery for a patient with bilateral breast cancer undergoing bilateral SSM with resection of the natural NAC. Bilateral reconstruction using breast implants and simultaneous nipple reconstruction was performed with a shorter recovery time than autologous transfer reconstruction, which significantly increased the patient's satisfaction (Figure 9).

In this study, overall patient satisfaction improved postoperatively. This may have been due to the fact that most complications could be treated with conservative management and that even major complications were accepted as the patients had been counseled thoroughly preoperatively.

This study also has its own limitations, which includes the small number of patients in the nipple-sparing group and the lack of solution related to the blood supply of the breast skin flap presented before or during surgery. The use of a single technique (inverted T reduction mammoplasty) is also a limitation of this study. Therefore, the classification and analysis of different surgical methods based on indication and postoperative scar treatments are necessary to achieve more favorable outcomes. The Breast-Q questionnaire used for surveying patient satisfaction deleted several items considering Asian oriental emotions and hence it could have caused selection bias on the result. In this study, the surgery algorithm was divided into partial and total mastectomy routes, which were comparatively analyzed based on postoperative patient satisfaction. However, more objective indicators should be used in future studies.

## 5. Conclusion

Reduction mammoplasty with inverted T incision was performed concomitantly with partial or nipple-sparing mastectomy in patients with breast cancer with large and/or ptotic breasts who desire an aesthetic change in the breast shape resulting in favorable patient satisfaction.

## Figures and Tables

**Table 1:** Patient demographics.

	Partial mastectomy (PM)	Nipple or skin mastectomy (NSSM)	*P* value
Number of patients	57	13	
Age (years)	45.18 ± 11.05	51.62 ± 8.96	0.0303
BMI (kg/m^2^)	26.74 ± 3.53	26.91 ± 4.34	0.9008

Preoperative volume of			
Right breast (cc)	663.85 ± 28.12	624.17 ± 98.52	0.5510
Left breast (cc)	664.32 ± 37.13	562.31 ± 80.81	0.2443

Diagnosis			
IDC	42 (73.7%)	10 (76.9%)	
DCIS	14 (24.6%)	3 (23.1%)	
Mucinous carcinoma	1 (1.8%)	0 (0%)	
Specimen weight (g)	177.74 ± 213.93	618.05 ± 338.17	<0.001

Axillary surgery			
SLNB	53 (93.0%)	11 (84.6%)	
ALND	4 (7%)	2 (15.4%)	

Cancer stage			
0/I/II/III	18 (31.6%)/22 (38.6%)/3 (5.3%)/0 (0%)	3 (23.1%)/5 (38.5%)/2 (15.4%)/0 (0%)	

Radiotherapy			
Neo/adjuvant/none	0 (0%)/51 (89.5%)/5 (8.8%)	0 (0%)/6 (46.2%)/7 (53.8%)	

Chemotherapy			
Neo/adjuvant/none	8 (14.0%)/23 (40.3%)/24 (42.1%)	5 (38.5%)/3 (23.1%)/6 (46.2%)	

BMI, body mass index; IDC, intraductal carcinoma; DCIS, ductal carcinoma in situ; SLNB, sentinel lymph node biopsy; ALND, axillary lymph node dissection. Data are presented as mean ± standard deviation or number (percentage).

**Table 2:** Postoperative complications.

	Partial mastectomy (*n* = 57)	Nipple or sparing mastectomy (*n* = 13)	*P* value
Seroma	6 (10.5%)	3 (23.1%)	<0.001
T Junction necrosis (minor/major)	5 (8.8%)/3 (5.3%)	4 (30.1%)/2 (15.4%)	0.0624/0.0511
NAC necrosis (partial/total)	3 (5.3%)/0	3 (23.1%)/3 (23.1%)	0.0363/0.0102
Fat necrosis	4 (7.0%)	0	0.0845
Infection	0	0	0.0271
Hematoma	3 (5.3%)	3 (23.1%)	0.7310

NAC, nipple-areolar complex. Data are presented as numbers (percentages).

**Table 3:** Patient satisfaction questionnaire.

	Preoperative score	Postoperative score	*P*value
Physical well-being			
The symmetry of the unclothed breast	3.9 ± 0.4	4.0 ± 0.2	0.0032
The shape of the unclothed breast	3.2 ± 0.3	4.1 ± 0.2	0.0426
The shape of the nipple-areolar complex	3.5 ± 0.1	4.0 ± 0.3	<0.001

Psychosocial well-being			
Natural breast shape with clothing	3.5 ± 0.2	4.3 ± 0.1	0.0310
Bra comfort	3.4 ± 0.4	4.2 ± 0.4	0.3137
Ability to wear fitted clothing	3.3 ± 0.3	4.3 ± 0.3	0.0292

Sexual well-being			
Sexual confidence	3.6 ± 0.1	3.9 ± 0.2	0.0221
Attractiveness in the mirror	3.5 ± 0.3	4.0 ± 0.1	0.4477

The satisfaction scores are presented as mean ± standard deviation on a 1–5 scale with 5 representing extremely satisfied.

## Data Availability

The data supporting the current study are given in the article.
